# Automated segmentation of the individual branches of the carotid arteries in contrast-enhanced MR angiography using DeepMedic

**DOI:** 10.1186/s12880-021-00568-6

**Published:** 2021-02-27

**Authors:** Magnus Ziegler, Jesper Alfraeus, Mariana Bustamante, Elin Good, Jan Engvall, Ebo de Muinck, Petter Dyverfeldt

**Affiliations:** 1 Division of Cardiovascular Medicine, Department of Health, Medicine and Caring Sciences, Linköping University, Linköping, Sweden; 2 Center for Medical Image Science and Visualization (CMIV), Linköping University, Linköping, Sweden; 3 Department of Cardiology, Department of Health, Medicine and Caring Sciences, Linköping University, Linköping, Sweden; 4 Department of Clinical Physiology, Department of Health, Medicine and Caring Sciences, Linköping University, Linköping, Sweden

**Keywords:** Atherosclerosis, Carotid arteries, Magnetic resonance imaging, Contrast-enhanced, Segmentation, Deep learning

## Abstract

**Background:**

Non-invasive imaging is of interest for tracking the progression of atherosclerosis in the carotid bifurcation, and segmenting this region into its constituent branch arteries is necessary for analyses. The purpose of this study was to validate and demonstrate a method for segmenting the carotid bifurcation into the common, internal, and external carotid arteries (CCA, ICA, ECA) in contrast-enhanced MR angiography (CE-MRA) data.

**Methods:**

A segmentation pipeline utilizing a convolutional neural network (DeepMedic) was tailored and trained for multi-class segmentation of the carotid arteries in CE-MRA data from the Swedish CardioPulmonsary bioImage Study (SCAPIS). Segmentation quality was quantitatively assessed using the Dice similarity coefficient (DSC), Matthews Correlation Coefficient (MCC), F_2_, F_0.5_, and True Positive Ratio (TPR). Segmentations were also assessed qualitatively, by three observers using visual inspection. Finally, geometric descriptions of the carotid bifurcations were generated for each subject to demonstrate the utility of the proposed segmentation method.

**Results:**

Branch-level segmentations scored DSC = 0.80 ± 0.13, MCC = 0.80 ± 0.12, F_2_ = 0.82 ± 0.14, F_0.5_ = 0.78 ± 0.13, and TPR = 0.84 ± 0.16, on average in a testing cohort of 46 carotid bifurcations. Qualitatively, 61% of segmentations were judged to be usable for analyses without adjustments in a cohort of 336 carotid bifurcations without ground-truth. Carotid artery geometry showed wide variation within the whole cohort, with CCA diameter 8.6 ± 1.1 mm, ICA 7.5 ± 1.4 mm, ECA 5.7 ± 1.0 mm and bifurcation angle 41 ± 21°.

**Conclusion:**

The proposed segmentation method automatically generates branch-level segmentations of the carotid arteries that are suitable for use in further analyses and help enable large-cohort investigations.

## Background

Stroke is one of the leading causes of death and disability in the western world and is often secondary to the rupture of atherosclerotic plaques in the carotid bifurcation. Atherosclerosis develops asymptomatically until a potentially catastrophic event occurs. As a result, non-invasive imaging is of interest for tracking the progression of atherosclerosis in the carotid arteries and has the potential to be used for risk stratification or treatment decisions [1].

In this realm, magnetic resonance imaging (MRI) presents several opportunities. MRI can be used for generating geometric [2, 3], hemodynamic [4], and compositional information from the carotid arteries [5–8]. However, to extract this information, the vessels must be identified and delineated from the images. When performed manually, this is a difficult and time-consuming process and the amount of time required increases with further localization, for example, when segmenting each arterial branch, i.e. internal (ICA), external (ECA), and common carotid arteries (CCA). Examining large cohorts would not be feasible without a significant decrease in user input or complete automation of these tasks. In addition, inter- and intra-observer variability decreases the consistency of segmentations and suggests an area where automated approaches could improve on current practice [9].

Medical image segmentation has been performed using multiple techniques, including intensity-based strategies, active contours, atlases, and methods using machine learning. With respect to segmentation of the carotid bifurcation using MRI data, level-set methods [10], deformable tube models [11], region growing [12], and supervised classifiers [12, 13] have demonstrated success. However, semi- or fully-automated multi-class segmentation of the carotid bifurcation into its constituent branches remains a difficult task. This is likely because there is no single clear image feature (e.g. intensity gradient) that can be used to define the branching point, which prevents the use of methods such as region growing that rely on such image features and suggests methods that incorporate larger numbers of image features are necessary. The limited ability to semi- or fully-automatically segment the carotid bifurcation hinders the development of automated methods for detecting stenoses, measuring carotid geometry, measuring hemodynamics, and measuring the composition of the vessel wall or plaques [14]. Automatic and accurate segmentations could be used to accelerate these applications and enable large cohort analyses with minimal user interaction.

Recently, deep learning and convolutional neural networks (CNNs) have emerged as useful methods for medical image segmentation and processing [15, 16]. For example, DeepMedic is an 11-layer deep CNN originally built with a dual-pathway architecture for brain lesion segmentation [17]. The DeepMedic framework is also capable of multi-class segmentation in 3D [17]. In this work, the DeepMedic framework was selected for use and tailored for a novel application; namely, multi-class segmentation of the carotid bifurcation. Therefore, the primary aim of this work is to develop and validate a method for the automated, multi-class segmentation of the carotid bifurcation using a CNN.

As the carotid arteries are a common site for atherosclerosis, many investigations have sought to examine underlying factors that predispose this region to atherosclerotic development. One factor thought to have significant effect is the vessel’s geometry, as it is believed to shape the hemodynamics of the vessel, and therefore the atherosclerotic development [18–20]. The geometry of the carotid bifurcation could therefore be seen as a patient-specific risk marker. As a result, several geometric descriptors of the carotid bifurcation have been proposed, including: branch diameters and the ratios between them, the bifurcation angle, and the vessel tortuosity [21]. Therefore, a secondary aim of this study is to demonstrate the utility of the multi-class segmentations by quantifying carotid bifurcation geometry in a large cohort of subjects with asymptomatic atherosclerosis.

## Methods

### Data

3D Contrast-enhanced MR angiography (CE-MRA) data for 268 subjects was acquired, though 74 subjects were excluded from further analysis based on a visual quality-assessment of the CE-MRA volume. Suboptimal timing of the CE-MRA imaging relative to the arrival of the contrast bolus was the primary cause for exclusion. Therefore, 194 subjects with bilateral imaging were included, i.e. 388 carotid bifurcations. Subjects were between 50 and 64 years of age and had at least one asymptomatic carotid plaque of at least 2.7 mm, measured by ultrasound. Subjects were recruited as part of the Swedish CArdioPulmonary bioImage Study (SCAPIS) [22]. Research was performed in accordance with the Declaration of Helsinki, this study received ethical approval, and all participants gave written, informed consent.

**Fig. 1:**
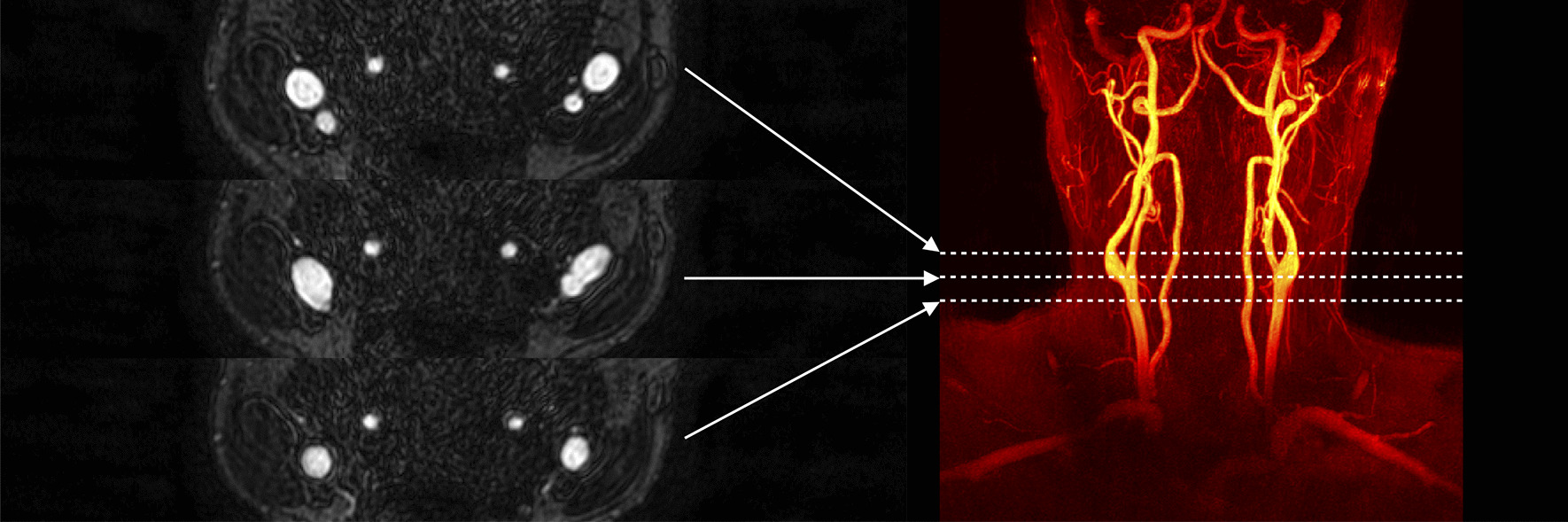
Typical CE-MRA images. The maximum intensity projection in the coronal plane is shown (right), with corresponding axial images (left) depicting the carotid arteries at the level of the CCA (bottom), bulb (middle), and after the bifurcation (top)

Imaging was performed using a 3 T Philips Ingenia scanner (Philips Healthcare, Best, the Netherlands) equipped with an 8-channel dedicated carotid coil (Shanghai Chenguang Medical Technologies, Shanghai, China). CE-MRA data was acquired post-injection of a gadolinium-based contrast agent (Gadovist, Bayer Schering Pharma AG) to generate bright-blood images for automated segmentations of the vessel lumen. A typical CE-MRA image is depicted in Fig. 1. Scan parameters included: a coronal slab with 3D field-of-view = 200 × 200 × 50 mm^3^ and matrix size 512 × 512 × 100, set to cover the carotid arteries from the clavicle to the circle of Willis, flip angle 27°, echo time 1.8 ms, repetition time 4.9 ms, parallel imaging (SENSE) factor 2, and a reconstructed spatial resolution of 0.48 × 0.48 × 0.50 mm^3^ [14, 22].

### Segmentation

The original DeepMedic network (version 0.7.0) and the tailored network were both implemented in Python 3.6.2 using Tensorflow v1.9.0, and MATLAB 2018a was used for post-processing.

### Preprocessing

Datasets were pre-processed to have a zero mean and unit variance. Images were subsequently divided in the sagittal plane and processed one side at a time, to simplify the segmentation task from 6 classes to 3.

### Network architecture and modifications from the original implementation

**Fig. 2:**
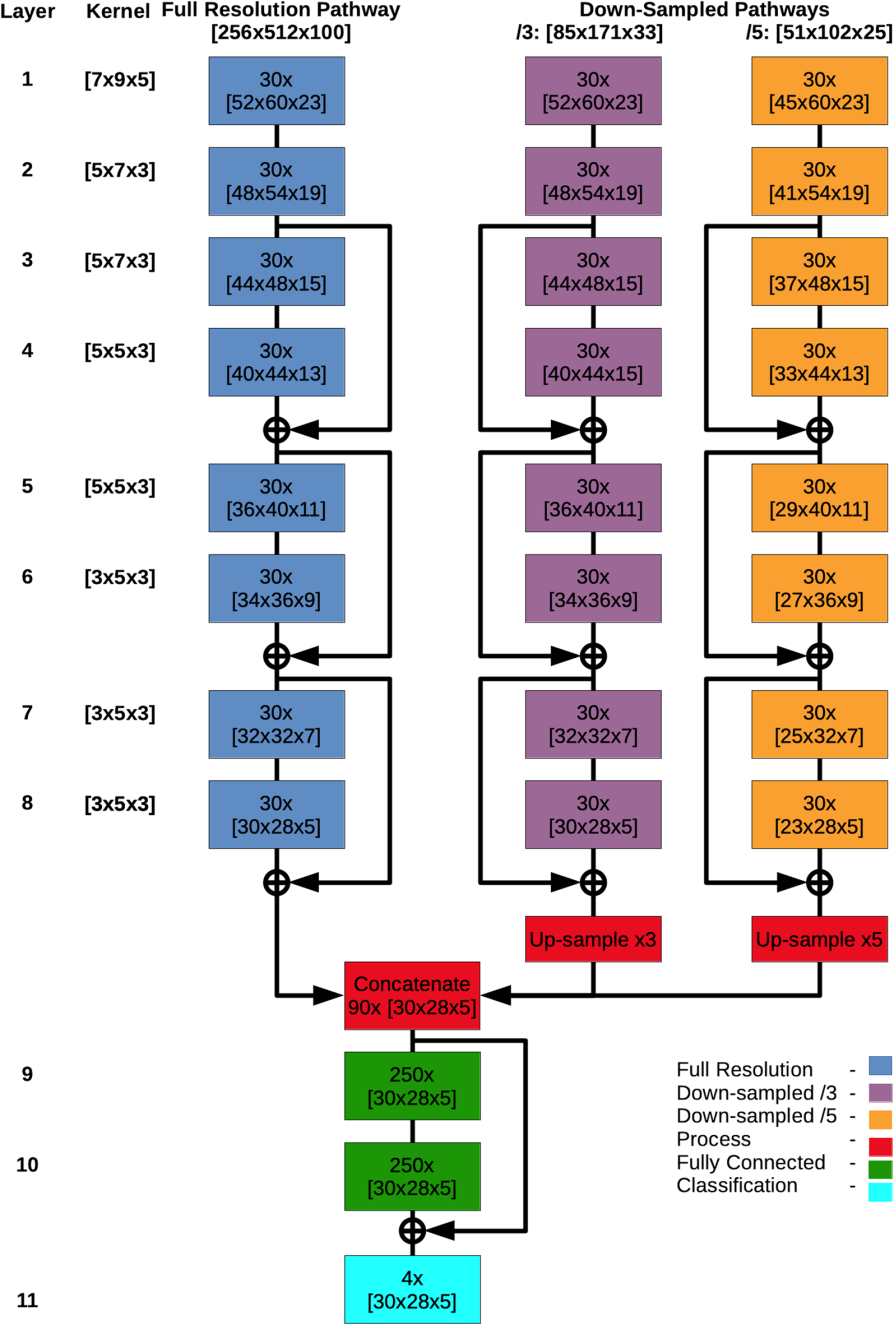
Architecture of proposed network. A complete listing of modified parameters is available in Additional Information 1

The proposed network is a modification of the original DeepMedic network as proposed by Kamnitsas et al. [17]. DeepMedic was originally proposed as a dual-pathway, 11-layer deep, 3D CNN for brain lesion segmentation. An overview of our modified network is seen in Fig. 2, and a full listing of modified parameters is available in Additional file: 1.

In our implementation of DeepMedic for multi-class segmentation of the carotid bifurcation, changes include the addition of another down-sampled pathway; an altered shape and increased size of kernels and input segments; a decrease in the number of feature maps in the deeper layers (layers 3–8); and, an increased number of neurons in the two fully-connected layers.

The additional sub-sampled pathway was added to expand on the spatial awareness idea discussed by Kamnitsas et al. [17]. By utilizing a full-resolution pathway, and pathways with sub-sampling factors of 3 and 5, the proposed network has an increased capability of capturing spatial context at multiple scales in the image volume and generating features that use this information [15]. This modification introduces additional computational cost compared to the original implementation.

The proposed network increases the receptive field by increasing the kernel size throughout the network. Moreover, while kernels in the original implementation are cubical, the proposed network uses kernels with a rectangular prism shape which is more representative of the non-cubic image volume, but also the tubular vessels that are the segmentation targets. These anisotropic and larger kernels may help the network learn more image features oriented to the shape of the carotid arteries. The increased kernel sizes also increase the computational cost compared to the original implementation.

### Training

Network training was performed on a workstation with a 3.6 GHz, 6-core processor with 64 GB RAM, and an NVIDIA Quadro 2000 GPU. The network was trained in 34 h using 20 epochs, with 52 bifurcations as training data and ten as validation. Table 1 shows an overview of data usage in this study. The ground-truth data used for training and testing was generated manually using ITK-SNAP [23] by two observers (M.Z., 5 years of experience in vascular MRI, E.G. 5 years of experience in vascular MRI) and had 3 non-background classes per side: the CCA, ECA, and ICA. Manual segmentation was performed by first manually labelling all voxels in the CCA, ECA, and ICA within a predefined distance from the flow divider (approximately 2.5 cm). Manual segmentations were post-processed to ensure no disjoint-regions or holes were present in the masks. Subsequently a centreline skeleton of this volume was created and the observer identified the centre of the carotid bifurcation (i.e. branch point), as well as the proximal CCA, and distal ECA and ICA points on the centrelines. Subsequently, the carotid bulb was divided based on this branch point and branch lengths were standardized to 2 cm.

**Table 1:** Total number of carotid bifurcations in each dataset, organized by activity

**DeepMedic—training phase**		**DeepMedic—evaluation phase**	
52	Training	336	Qualitative (observer scored)
10	Validation*	46	Quantitative (segmentation and geometric metrics)

**DeepMedic—utility demonstration—cohort geometric analysis**			
388	Quantification of diameter, diameter ratio, bifurcation angle		

^*^Validation dataset re-used in evaluation phase

The ${F}_{\beta }$ measure was used as the cost function, with $\beta =2$, to weigh the importance of specificity larger than sensitivity. The ${F}_{\beta }$ measure is defined as:

$$F_{\beta } = (1 + \beta^{2} )\frac{(specificity*sensitivity)}{{(\beta^{2} *specificity + sensitivity)}}\quad [24]$$

### Post-processing

The raw output of the proposed network was post-processed to remove any disjoint regions and increase the consistency of the class separation between the CCA, ICA, and ECA in the carotid bulb. Connected component analyses were used to identify any regions that were not connected to the largest component for their respective class. These regions were subsequently removed if they were not connected to another non-background class or merged with the connected non-background class. Additionally, morphological operations were used to increase the consistency of the class separation. Morphological opening and closing, using 2 × 2 × 2 kernels, were used to create a more uniform boundary between classes by smoothing the edges in the carotid bulb. Segmentations were standardized by cropping data outside a radius of 2 cm from the centre of the bifurcation.

### Geometric analysis

**Fig. 3:**
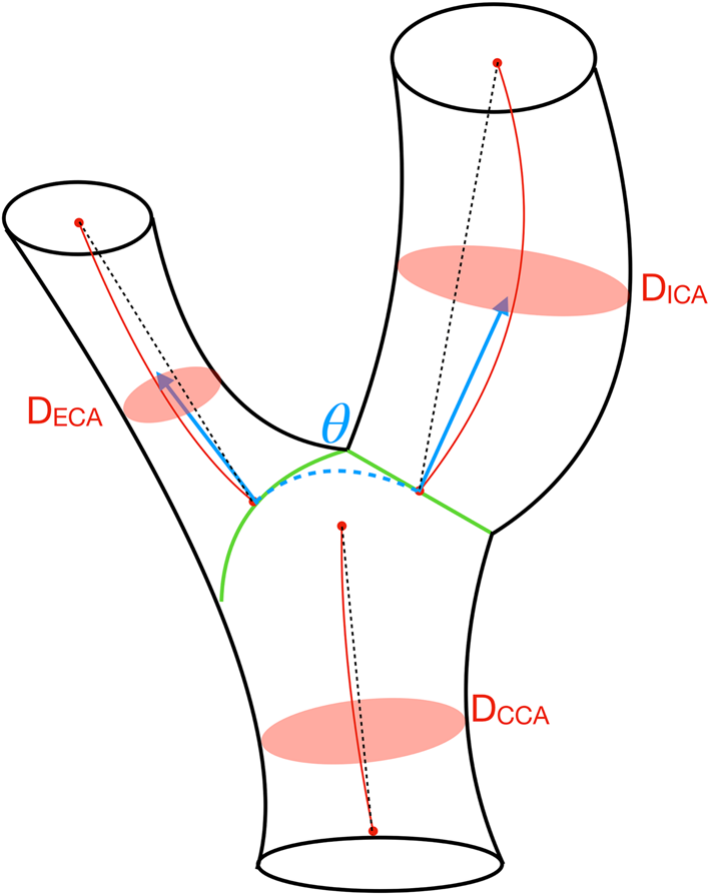
Schematic of the Carotid Artery Bifurcation. Green lines depict boundaries between vessel branches. Red solid lines depict centrelines through vessels. Black dotted lines depict straight-line paths between the start- and end-points of each vessel branch. Blue vectors mark the bifurcation angle ($\uptheta$)

Geometric analysis was performed using in-house software, developed in MATLAB. Several geometric descriptors were automatically computed to demonstrate the utility of the multi-class segmentations of the carotid bifurcation (Fig. 3):

- The mean diameter for each branch of the carotid bifurcation. The mean diameter was estimated by first calculating the diameter using planes normal to the centreline vector at each voxel along the centreline. For each of these planes, the diameter was calculated by assuming circularity and measuring the planar area within the segmentation.
- The ratio between respective downstream and upstream branch diameters (e.g. ICA/CCA).
- The bifurcation angle, which was calculated using vectors aligned with the centrelines of the ICA and ECA. Both vectors originated at the centreline-point nearest to the bifurcation on each branch and intersect with the midpoint of the centreline on their respective branches. Centrelines were smoothed.

### Evaluation

Segmentations generated by the proposed modification of the DeepMedic network were examined both quantitatively and qualitatively. Quantitatively, multi-class data for 46 bifurcations was compared against manually generated ground-truth (GT) data using the following metrics: Dice similarity coefficient (DSC), Matthews Correlation Coefficient (MCC) [25], F_2_, F_0.5_, and true positive ratio (TPR) (Table 1). Two-sample t-tests were used to determine whether or not there were differences in segmentation performance between branches, and between branch-specific and bifurcation segmentations using a Bonferroni adjusted significance level (α) of 0.0025 (0.05/20). A listing of statistical tests included for this Bonferroni correction factor is included in Additional file 1: Table S1. The difference in performance between the original implementation of DeepMedic and the proposed implementation, as well as the impact of the post-processing stage, were examined in a subset of 20 bifurcations using the DSC.

In addition, for all subjects outside the DeepMedic training cohort (i.e. 168 subjects, 336 bifurcations, Table 1), segmentations were examined qualitatively and the quality was assessed visually (MZ, 5 years of experience in vascular MRI). Qualitative scores were assigned per bifurcation using the following 0–4 scale, based on the amount of adjustments needed before the masks could be used for future analyses: 0—failed segmentation of vessel or one or more incorrect branch classifications; 1—major adjustments (i.e. 10 min) required; 2—substantial adjustments (i.e. 5 min) required; 3—minor adjustments (i.e. 2 min) required; and 4—no adjustments required. In addition, 25% of the cohort of carotid bifurcations available for qualitative assessment were randomly selected and evaluated by two additional observers (FV, and PD, 5 and 10 + years’ experience cardiac and vascular MRI, respectively). Fleiss Kappa (κ) was used to evaluate the agreement between the observers [26, 27].

Geometric descriptors of the carotid arteries generated using the GT segmentations and automatically generated segmentations were compared in the quantitative evaluation cohort of 46 bifurcations (Table 1). Two-sample t-tests were used to determine whether or not there were differences between branches using a Bonferroni-adjusted significance level (α) of 0.0083 (0.05/6). A listing of statistical tests included for this Bonferroni correction factor is included in Additional file 1: Table S1. Automatically generated segmentations were used to generate cohort-wide (n = 388 bifurcations, Table 1) descriptive statistics for carotid geometry, and this data is presented as mean ± standard deviation. The coefficient of variation was also used to describe the variation of a given descriptor within the cohort and was calculated as (standard deviation/mean).

## Results

Total analysis time, including pre-processing, segmentation using the proposed method, post-processing, and evaluation against ground truth was approximately four minutes per bifurcation. Segmentation time (i.e. inference time) was approximately 45 s per bifurcation. Manual segmentation time was approximately 15–20 min per bifurcation.

**Fig. 4:**
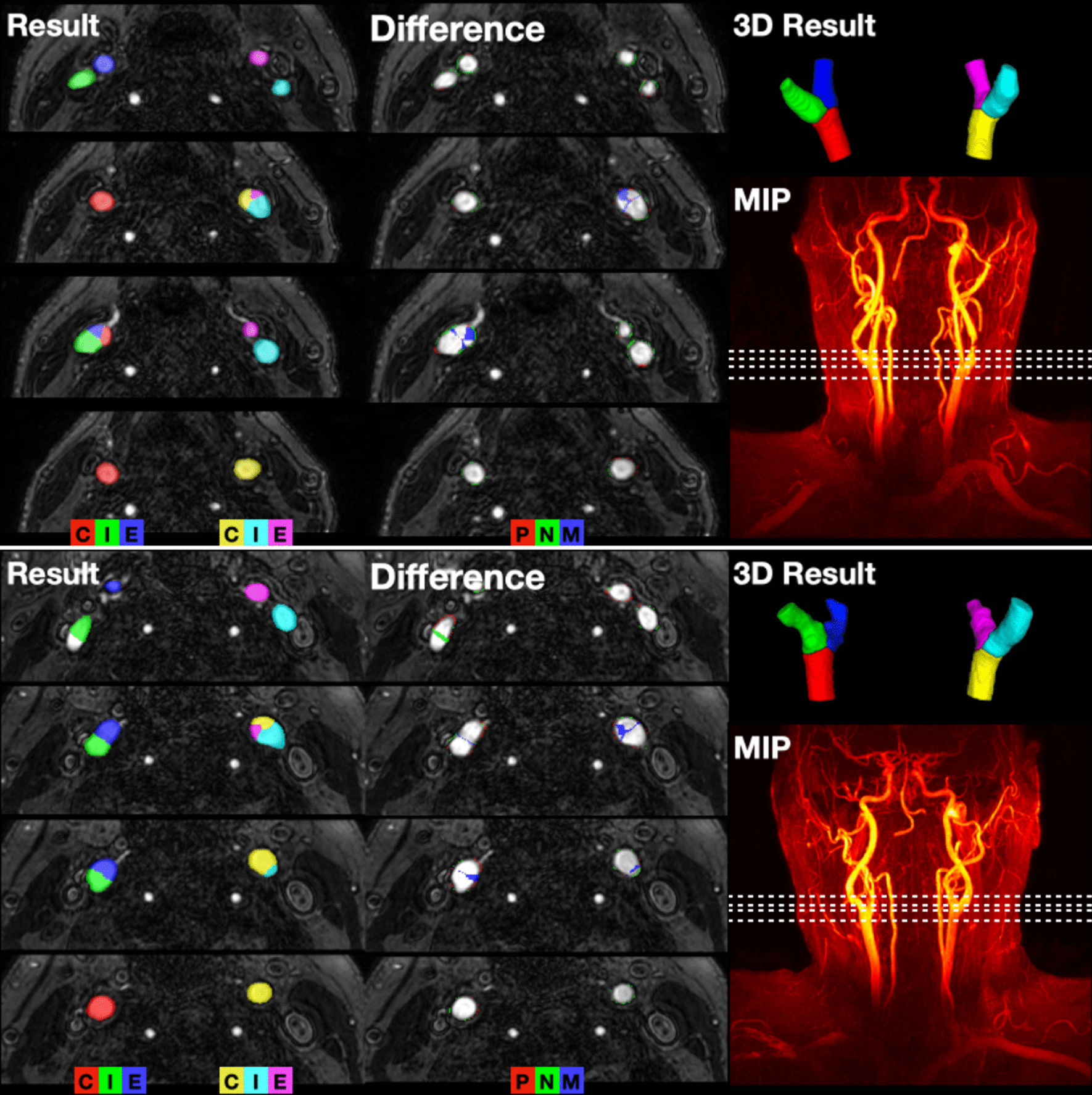
Example segmentation result for two subjects (upper and lower panels). Left panel depicts segmentation result overlaid on four axial CE-MRA slices, middle panel depicts the difference between GT and result, right panel depicts 3D surface visualization of result and maximum intensity projection of subject with axial slice locations indicated. C-CCA; I-ICA; E-ECA; P-False Positive; N-False Negative; M-class mismatch. Class mismatch indicates voxel was in both result and GT, but the voxel in question was assigned to a different class

A summary of the different quantitative metrics for comparison against GT is presented in Table 2. For a given branch (i.e. CCA, ICA, or ECA), the DSC was 0.80 ± 0.13, MCC = 0.80 ± 0.12, F_2_ = 0.82 ± 0.14, F_0.5_ = 0.78 ± 0.13, and TPR = 0.84 ± 0.16. Performance was good considering these quantitative metrics, and no statistically significant differences between segmentation performance between branches were found when comparing the CCA and ECA using the DSC, MCC, and F_2_ metrics and the between the CCA and ICA using the F_2_ and TPR metrics. In this cohort of 23 subjects, or 138 branches, 5 had a DSC or MCC score of less than 0.5. Examining the whole-bifurcation segmentations showed improved results, e.g. DSC = 0.85 ± 0.07, as potential disagreement between the multi-class result and GT in the carotid bulb is not considered. No statistically significant differences were found between whole-bifurcation and branch-specific segmentation quality scores for DSC, MCC, F_2_, and F_0.5_. Figure 4 depicts two representative segmentation results from the testing cohort. A complete listing of results for each subject can be found in Additional file 1: Tables S2–S6, and a summary of the statistical testing is shown in Additional file 1: Table S7. Example segmentations with poorer performance can be found in Additional file 1: Figure S1.

**Table 2:** Summary of segmentation quality metrics for testing cohort

Metric	Region							
	Left	LCCA	LICA	LECA	Right	RCCA	RICA	RECA
DSC	0.86 ± 0.07	0.82 ± 0.10	0.78 ± 0.17	0.78 ± 0.11	0.85 ± 0.07	0.84 ± 0.06	0.80 ± 0.15	0.77 ± 0.18
MCC	0.86 ± 0.07	0.83 ± 0.09	0.79 ± 0.16	0.79 ± 0.11	0.85 ± 0.07	0.84 ± 0.05	0.81 ± 0.14	0.77 ± 0.18
F_2_	0.89 ± 0.09	0.85 ± 0.10	0.79 ± 0.19	0.80 ± 0.14	0.87 ± 0.09	0.87 ± 0.06	0.82 ± 0.18	0.80 ± 0.19
F_0.5_	0.84 ± 0.08	0.80 ± 0.12	0.78 ± 0.16	0.77 ± 0.10	0.84 ± 0.09	0.81 ± 0.08	0.80 ± 0.12	0.74 ± 0.18
TPR	0.91 ± 0.10	0.88 ± 0.12	0.80 ± 0.22	0.82 ± 0.16	0.89 ± 0.12	0.90 ± 0.08	0.83 ± 0.20	0.83 ± 0.21

Values are presented as mean ± SD (n = 23 subjects)

Compared to the original implementation of DeepMedic, the proposed implementation that is tailored for segmentation of the carotid arteries displayed improvement, both before and after post-processing, considering all regions (Additional file 1: Table S8). Excluding the effects of post-processing, the proposed implementation of DeepMedic increased the DSC metric by 0.13, on average. When postprocessing was used, the proposed implementation returns DSC scores improved by 0.04 on average.

Qualitative assessment was performed for all subjects outside of the training cohort (i.e. 168 subjects, 336 bifurcations). The median and modal score per bifurcation was 4. The majority of bifurcations, 61.3%, were judged to be suitable for further analyses without adjustment. Table 3 summarizes the results of qualitative assessment. Interobserver analysis considering 25% of the available bifurcations indicated that interobserver agreement was modest (κ = 0.11, *p* = 0.003), and on average bifurcations received a score of 3. The median and modal scores in this subset were 3 and 4, respectively.

**Table 3:** Qualitative assessment of segmentations for subjects outside the training cohort (n = 336)

Qualitative score	Count	% of Total
0—Fail	16	4.8
1—Major adjustments required	7	2.1
2—Substantial adjustments required	22	6.6
3—Minor adjustments required	85	25.3
4—No adjustments required	206	61.3

Demonstrating the utility of the segmentations generated by the proposed network, geometric descriptors automatically derived from all segmentations are presented in Table 4. Geometric descriptors derived from both the GT and automatically generated segmentations in the testing cohort showed minor differences (Table 5).

**Table 4:** Results of cohort-wide geometric analyses (n = 388 bifurcations)

Parameter	Value	Coefficient of variation
Diameter CCA [mm]	8.7 ± 1.1	0.13
Diameter ICA [mm]	7.5 ± 1.4	0.19
Diameter ECA [mm]	5.7 ± 1.0	0.17
Diameter ratio ICA/CCA [–]	0.88 ± 0.15	0.17
Diameter ratio ECA/CCA [–]	0.67 ± 0.09	0.14
Bifurcation angle [°]	49.5 ± 13.4	0.27

All parameters are listed as mean ± SD

**Table 5:** Comparison of geometric parameters generated from manually and automatically generated segmentations (n = 46 bifurcations)

Parameter	Difference (Manual–Automatic)	*p*-value
Diameter CCA [mm]	− 0.69 ± 0.91*	1.8e−3
Diameter ICA [mm]	− 0.27 ± 1.30	0.852
Diameter ECA [mm]	− 0.39 ± 0.82	0.319
Diameter ratio ICA/CCA [–]	0.04 ± 0.14	0.259
Diameter ratio ECA/CCA [–]	0.05 ± 0.09	0.012
Bifurcation angle [°]	− 2.6 ± 25.5	0.506

^*^Statistically Significant difference (e.g. *p* < α = 0.0083)

## Discussion

In this work, we introduced a novel application of deep learning and demonstrated multi-class segmentation of the carotid arteries in CE-MRA into its constituent branches. Segmentations scored highly in both quantitative and qualitative evaluations, and their utility was demonstrated by the automated quantification of carotid bifurcation geometry.

Quantitative evaluation of the proposed segmentation method using the DSC, MCC, F_2_, F_0.5_, and TPR metrics indicates that the method has high performance and low failure rates making it acceptable for further use. Segmentations were evaluated on both a branch-level, and whole-bifurcation-level in 46 bifurcations. Considering the branch-level segmentations, we found that a given branch segmentation (i.e. CCA, ICA, or ECA) had scores that were on average acceptable for further use (DSC, MCC, F_2_, and TPR > 0.8). Moreover, performance was relatively stable with respect to branch. In this testing cohort of 46 bifurcations, or 138 branches, only five had unacceptably low DSC or MCC scores (< 0.5). Three such branches were confined to a single subject (#6, Additional file 1: Tables S2–S6), and all were located in the ICA or ECA. The mean scores for the CCA were on average higher than the ICA and ECA branches, although these differences were not significant for all tests (Additional file 1: Table S7). This may be explained by the ICA and ECA being smaller and having more anatomical variation than the CCA.

The bifurcation-level segmentations on average scored higher than the multi-class segmentation of the bifurcation into constituent branches. This is likely related to the carotid bulb region where the branches meet. Dividing the carotid bulb into constituent branches is a difficult task for observers to perform manually as there is no signal intensity difference and bifurcation geometry has wide variation. Therefore, to create GT data, observers segmented the entire bifurcation region first and relied on the branch-point of centrelines to divide the CA. This may induce variation in how this region is subdivided and likely contributes to the lower scores. Increasing the data available for training the network could improve performance in this aspect.

Visual inspection of the segmentations (Table 2) indicates that the proposed segmentation method has the capability to dramatically reduce the time needed to produce high quality segmentations of the carotid bifurcation. In 336 bifurcations, we found that the majority (61.3%) did not need any adjustment before they could be used for analyses. A further 25.3% of bifurcations needed only minor adjustments that could be performed in less than two minutes. Inter-observer comparisons in 25% of the subjects available for qualitative assessment verified this result and therefore strengthened the notion that the proposed method has the ability to dramatically reduce the time needed to produce segmentations suitable for analyses, even if minor corrections may be sometimes necessary.

To demonstrate the utility of the segmentation framework, we derived common geometric descriptors of carotid bifurcation geometry for the entire cohort. In a study cohort that includes both men and women between 50 and 65 years of age with carotid atherosclerosis, the expected range of carotid geometry is large. For example, we found that the bifurcation angle had a large coefficient of variation (51%), though this was in line with previously reported values [20]. Previously reported values for CCA and ICA diameter were smaller than our findings [28], though that cohort was younger and measurements were performed by ultrasound. Comparing the geometric descriptors as derived from GT and automatically generated segmentations in the testing cohort showed good correspondence, though differences were found, particularly in the diameter of the CCA. However, this difference in diameter is minor, on the order of one voxel. The large geometric variation of carotid bifurcation anatomy, as quantified here, may also indicate the need for a substantially larger training set to achieve improved segmentation results. A larger training set should also include subjects with more severe atherosclerosis.

This study has several limitations. With limited amounts of multi-class GT data available for the carotid bifurcation, owing to the time-consuming nature of creating this data (15–20 min per bifurcation), the data available for training and testing was relatively small. Therefore, post-processing plays an important role in the success of the proposed segmentation pipeline and helps compensate for the limited amount of ground-truth data. With more ground-truth data used for training and testing, the importance of post-processing stages may be reduced as the network increases in performance. That being said, the network performs strongly even with the relatively limited training set, and shows that deep learning based methods are applicable even with less-than-ideal datasets. In addition, the difficulty of dividing the carotid bulb into constituent branches likely induces undesirable variation in the GT data. This study also lacks an inter- and intra-observer variability study with respect to the generation of ground-truth data. Finally, this study had limited comparisons to previously published methods, both traditional and deep learning based, as comparable methods for automated multi-class segmentation methods for the carotid bifurcation are lacking. Therefore, we cannot conclude that the proposed method is wholly superior, and alternative approaches may outperform the proposed method.

In conclusion, a CNN was applied to segment the carotid bifurcation into its constituent branches and facilitate future analyses. Segmentations scored well in both quantitative and qualitative analyses, and to demonstrate the utility of these segmentations we automatically generated geometric descriptions for 388 carotid bifurcations. This segmentation method demonstrated the ability to accelerate analyses by dramatically reducing the amount of manual labour required, enabling large cohort studies.

## Supplementary Information

**Additional file 1.** DeepMedic configuration. **Additional file 1: Table S1.** DSC scores. **Table S2.** MCC scores. **Table S3.** F_2 scores. **Table S4.** F_0.5 scores. **Table S5.** TPR scores. **Table S6.** Summary of statistical tests. **Table S7.** Comparison between original and proposed DeepMedic Configurations before and after post-processing. **Figure S1.** Example unacceptable segmentation results.

## Data Availability

The MRI datasets generated and analysed during the current study are not publicly available due to confidentiality agreements but are available from the corresponding author and Linköping University Hospital on reasonable request for researchers who meet the criteria for access to confidential data. The institutional review board form states that the data obtained from the patients will be stored on secure computers within the Linköping University Hospital.

## Abbreviations

CCA: Common carotid artery
CE: Contrast-enhanced
CNN: Convolutional neural network
DSC: Dice similarity coefficient
ECA: External carotid artery
GPU: Graphics processing unit
GT: Ground truth
ICA: Internal carotid artery
MCC: Matthews correlation coefficient
TPR: True positive ratio
