# Supplementary material for: Automated segmentation of the individual branches of the carotid arteries in contrast-enhanced MR angiography using DeepMedic
Source: BMC Med Imaging. 2021 Feb 27;21:38. doi: 10.1186/s12880-021-00568-6 (PMC7912466; doi:10.1186/s12880-021-00568-6)
Supplement: Supplementary file 1 — Additional file 1. DeepMedic configuration. Additional file 1: Table S1. DSC scores. Table S2. MCC scores. Table S3. F_2 scores. Table S4. F_0.5 scores. Table S5. TPR scores. Table S6. Summary of statistical tests. Table S7. Comparison between original and proposed DeepMedic Configurations before and after post-processing. Figure S1. Example unacceptable segmentation results. [file 12880_2021_568_MOESM1_ESM.docx]

**Supplementary Information for:**

**Automated Segmentation of the Individual Branches of the Carotid Arteries in Contrast-Enhanced MR Angiography using DeepMedic**

Magnus Ziegler PhD^1,2,*^, Jesper Alfraeus MSc^1^, Mariana Bustamante PhD^1,2^, Elin Good MD^1,2,3^, Jan Engvall MD PhD^1,2,4^, Ebo de Muinck MD PhD^1,2,3^ and Petter Dyverfeldt PhD^1,2^

# Additional Information

## **Additional file 1. DeepMedic Configuration**

**Training Configuration**

- Training Data: CEMRA data for 52 carotid bifurcations from 26 patients

- Sampling type: centre voxel starting on background or foreground with a probability of 0.5

- Number of epochs: 20, with 15 sub-epochs per epoch

- Data Augmentation, reflect: the data is reflected with 50% probability in the sagittal plane.

- Data Augmentation, intensity: The variables shift are sampled from a gaussian distribution, the mean and standard deviation of the two are shifted: [0.0, 0.1], multi: [1.0, 0.05]. This is applied to the image in order to get a final image intensity of I', where:
 I' = (I+shift)*multi.

- Learning Rate: starting at 0.001, decreased by factor 2 on epochs [8,11,14,16,18].

- Optimizer: RmsProp with rho = 0.9 and epsilon = 10e-4

- Regularization: L1 with a factor 10e-6 and L2 with factor 10e-4.

**Model Configuration**

Normal Pathway:

- Number of output classes: 4, 3 per vessel and 1 background

- Number of feature maps per layer: [30,30,30,30,30,30,30,30]

- Kernel dimensions per layer: [[7,9,5], [5,7,3], [5,7,3], [5,5,3], [5,5,3], [3,5,3], [3,5,3], [3,5,3]]

- Layers with residual connections - [4,6,8]

Subsampled Pathways:

- Number of feature maps per layer - [30,30,30,30,30,30,30,30]

- Kernel dimensions per layer - [[7,9,5], [5,7,3], [5,7,3], [5,5,3], [5,5,3], [3,5,3], [3,5,3], [3,5,3]]

- Layers with residual connections} - [4,6,8]

- Subsample factor - [[3,3,3], [5,5,5]]

Fully Connected Layers:

- Number of feature maps per layer: [250,250]

- Kernel dimensions for the first fully connected layer: [3,3,3]

- Residual connections for the fully connected hidden layers: [2]

Size of Image Segments

- Training: [58,68,25]

- Validation: [29,41,19]

- Testing: [40,65,25]

Batch Sizes

- Training: [8]

- Validation: [8]

- Testing: [8]

Miscellaneous

- Dropout rates for the fully connected layers: [0.0,0.6,0.6]

- Kernel weight initialization: kernel weights were initialized by sampling from the normal distribution N(0,$\sqrt{\frac{2}{n_{I}^{in}}}$.)

- Activation function: parametric rectified linear unit (PReLU)

- Batch normalization: rolling average over 60 batches.

- kernels used stride 1

- padding used zeros and was sized to yield full convolutions

## **Additional file 1: Table S1. Statistical tests per Bonferroni grouping**

Each numbered item represents a single t-test. Tests were grouped by parameter theme and the total number of tests per group was used in the Bonferroni Correction. i.e. 6 geometric tests yields corrected p-value of (0.05/6) = 0.0083.

| Group 1: Segmentation Quality Metrics | Group 2: Geometric Parameters (Automatic vs Manual) |
| --- | --- |
| 1. Bifurcation vs Branches (DSC) | 1) Diameter, CCA |
| 1. Bifurcation vs Branches (MCC) | 2) Diameter ICA |
| 1. Bifurcation vs Branches (F_2) | 3) Diameter ECA |
| 1. Bifurcation vs Branches (F_0.5) | 4) Diameter Ratio ICA/CCA |
| 1. Bifurcation vs Branches (TPR) | 5) Diameter Ratio ECA/CCA |
| 1. CCA vs ICA (DSC) | 6) Bifurcation Angle |
| 1. CCA vs ICA (MCC) |  |
| 1. CCA vs ICA (F_2) |  |
| 1. CCA vs ICA (F_0.5) |  |
| 1. CCA vs ICA (TPR) |  |
| 1. CCA vs ECA (DSC) |  |
| 1. CCA vs ECA (MCC) |  |
| 1. CCA vs ECA (F_2) |  |
| 1. CCA vs ECA (F_0.5) |  |
| 1. CCA vs ECA (TPR) |  |
| 1. ICA vs ECA (DSC) |  |
| 1. ICA vs ECA (MCC) |  |
| 1. ICA vs ECA (F_2) |  |
| 1. ICA vs ECA (F_0.5) |  |
| 1. ICA vs ECA (TPR) |  |

## **Additional file 1: Table S2**. DSC

**Dice Similarity Coefficient (DSC)**

*** -** Sum Score of Left and Right CCA, ICA, and ECA. A score of 6 represents a perfect score.

| **Subject** | **Left CA** | **LCCA** | **LICA** | **LECA** | **Right CA** | **RCCA** | **RICA** | **RECA** | **Sum Score*** | **Rank** |
| --- | --- | --- | --- | --- | --- | --- | --- | --- | --- | --- |
| **1** | 0.89 | 0.88 | 0.82 | 0.59 | 0.81 | 0.90 | 0.81 | 0.84 | 4.84 | 10 |
| **2** | 0.92 | 0.82 | 0.86 | 0.46 | 0.82 | 0.83 | 0.81 | 0.77 | 4.54 | 21 |
| **3** | 0.90 | 0.89 | 0.84 | 0.87 | 0.89 | 0.86 | 0.90 | 0.84 | 5.21 | 6 |
| **4** | 0.88 | 0.74 | 0.81 | 0.74 | 0.86 | 0.83 | 0.85 | 0.81 | 4.78 | 14 |
| **5** | 0.94 | 0.93 | 0.91 | 0.90 | 0.94 | 0.92 | 0.92 | 0.91 | 5.49 | 1 |
| **6** | 0.63 | 0.55 | 0.15 | 0.67 | 0.64 | 0.75 | 0.37 | 0.00 | 2.49 | 23 |
| **7** | 0.83 | 0.87 | 0.82 | 0.78 | 0.86 | 0.80 | 0.78 | 0.77 | 4.81 | 12 |
| **8** | 0.83 | 0.76 | 0.78 | 0.77 | 0.80 | 0.78 | 0.83 | 0.77 | 4.68 | 15 |
| **9** | 0.78 | 0.90 | 0.86 | 0.89 | 0.91 | 0.79 | 0.84 | 0.55 | 4.84 | 11 |
| **10** | 0.88 | 0.78 | 0.77 | 0.81 | 0.83 | 0.87 | 0.85 | 0.83 | 4.90 | 9 |
| **11** | 0.84 | 0.86 | 0.83 | 0.73 | 0.91 | 0.85 | 0.72 | 0.69 | 4.68 | 17 |
| **12** | 0.86 | 0.65 | 0.66 | 0.89 | 0.79 | 0.84 | 0.84 | 0.80 | 4.68 | 16 |
| **13** | 0.91 | 0.94 | 0.89 | 0.83 | 0.93 | 0.85 | 0.84 | 0.74 | 5.10 | 7 |
| **14** | 0.95 | 0.91 | 0.91 | 0.88 | 0.92 | 0.94 | 0.94 | 0.90 | 5.48 | 2 |
| **15** | 0.92 | 0.92 | 0.91 | 0.91 | 0.95 | 0.83 | 0.90 | 0.87 | 5.35 | 4 |
| **16** | 0.86 | 0.86 | 0.77 | 0.79 | 0.88 | 0.83 | 0.83 | 0.72 | 4.80 | 13 |
| **17** | 0.77 | 0.71 | 0.48 | 0.67 | 0.75 | 0.78 | 0.39 | 0.80 | 3.83 | 22 |
| **18** | 0.82 | 0.84 | 0.69 | 0.77 | 0.80 | 0.78 | 0.67 | 0.82 | 4.56 | 19 |
| **19** | 0.94 | 0.77 | 0.86 | 0.71 | 0.83 | 0.90 | 0.95 | 0.91 | 5.09 | 8 |
| **20** | 0.92 | 0.87 | 0.89 | 0.89 | 0.91 | 0.91 | 0.91 | 0.86 | 5.33 | 5 |
| **21** | 0.94 | 0.95 | 0.92 | 0.87 | 0.94 | 0.89 | 0.93 | 0.89 | 5.45 | 3 |
| **22** | 0.80 | 0.76 | 0.70 | 0.78 | 0.78 | 0.70 | 0.79 | 0.81 | 4.55 | 20 |
| **23** | 0.79 | 0.80 | 0.75 | 0.80 | 0.81 | 0.78 | 0.79 | 0.73 | 4.65 | 18 |
| **Mean** | 0.86 | 0.82 | 0.78 | 0.78 | 0.85 | 0.84 | 0.80 | 0.77 | 4.79 |  |
| **sd** | 0.07 | 0.10 | 0.17 | 0.11 | 0.07 | 0.06 | 0.15 | 0.18 | 0.62 |  |

## **Additional file 1: Table S3. MCC**

**Matthews Correlation Coefficient (MCC)**

*** -** Sum Score of Left and Right CCA, ICA, and ECA. A score of 6 represents a perfect score.

| **Subject** | **Left CA** | **LCCA** | **LICA** | **LECA** | **Right CA** | **RCCA** | **RICA** | **RECA** | **Sum Score*** | **Rank** |
| --- | --- | --- | --- | --- | --- | --- | --- | --- | --- | --- |
| **1** | 0.89 | 0.88 | 0.83 | 0.59 | 0.81 | 0.90 | 0.82 | 0.85 | 4.87 | 10 |
| **2** | 0.92 | 0.82 | 0.86 | 0.47 | 0.82 | 0.84 | 0.82 | 0.78 | 4.57 | 21 |
| **3** | 0.90 | 0.90 | 0.85 | 0.88 | 0.90 | 0.86 | 0.90 | 0.85 | 5.23 | 6 |
| **4** | 0.88 | 0.76 | 0.82 | 0.74 | 0.87 | 0.84 | 0.85 | 0.81 | 4.83 | 14 |
| **5** | 0.94 | 0.93 | 0.91 | 0.90 | 0.94 | 0.92 | 0.92 | 0.91 | 5.49 | 1 |
| **6** | 0.64 | 0.55 | 0.16 | 0.68 | 0.64 | 0.76 | 0.38 | 0.00 | 2.53 | 23 |
| **7** | 0.83 | 0.87 | 0.82 | 0.79 | 0.86 | 0.80 | 0.79 | 0.77 | 4.83 | 13 |
| **8** | 0.84 | 0.78 | 0.79 | 0.78 | 0.81 | 0.80 | 0.83 | 0.78 | 4.76 | 15 |
| **9** | 0.78 | 0.90 | 0.87 | 0.89 | 0.92 | 0.80 | 0.84 | 0.56 | 4.86 | 11 |
| **10** | 0.88 | 0.79 | 0.78 | 0.81 | 0.84 | 0.88 | 0.85 | 0.83 | 4.94 | 9 |
| **11** | 0.85 | 0.86 | 0.83 | 0.74 | 0.91 | 0.85 | 0.73 | 0.72 | 4.74 | 17 |
| **12** | 0.87 | 0.66 | 0.69 | 0.89 | 0.80 | 0.85 | 0.84 | 0.81 | 4.73 | 18 |
| **13** | 0.91 | 0.94 | 0.90 | 0.83 | 0.93 | 0.85 | 0.84 | 0.75 | 5.11 | 7 |
| **14** | 0.95 | 0.91 | 0.91 | 0.88 | 0.93 | 0.94 | 0.94 | 0.90 | 5.49 | 2 |
| **15** | 0.92 | 0.92 | 0.92 | 0.92 | 0.95 | 0.83 | 0.90 | 0.87 | 5.36 | 4 |
| **16** | 0.86 | 0.87 | 0.78 | 0.79 | 0.88 | 0.83 | 0.84 | 0.74 | 4.84 | 12 |
| **17** | 0.77 | 0.73 | 0.54 | 0.68 | 0.75 | 0.79 | 0.46 | 0.80 | 4.00 | 22 |
| **18** | 0.82 | 0.85 | 0.71 | 0.79 | 0.81 | 0.78 | 0.67 | 0.82 | 4.63 | 20 |
| **19** | 0.94 | 0.77 | 0.86 | 0.72 | 0.83 | 0.90 | 0.95 | 0.91 | 5.10 | 8 |
| **20** | 0.92 | 0.87 | 0.89 | 0.89 | 0.91 | 0.91 | 0.91 | 0.86 | 5.33 | 5 |
| **21** | 0.94 | 0.95 | 0.92 | 0.88 | 0.94 | 0.90 | 0.93 | 0.89 | 5.45 | 3 |
| **22** | 0.81 | 0.78 | 0.73 | 0.79 | 0.80 | 0.73 | 0.80 | 0.81 | 4.64 | 19 |
| **23** | 0.80 | 0.81 | 0.77 | 0.81 | 0.82 | 0.79 | 0.80 | 0.75 | 4.74 | 16 |
| **Mean** | 0.86 | 0.83 | 0.79 | 0.79 | 0.85 | 0.84 | 0.81 | 0.77 | 4.83 |  |
| **sd** | 0.07 | 0.09 | 0.16 | 0.11 | 0.07 | 0.05 | 0.14 | 0.18 | 0.60 |  |

## **Additional file 1: Table S4. F_2**

**F-Beta, Beta = 2**

*** -** Sum Score of Left and Right CCA, ICA, and ECA. A score of 6 represents a perfect score.

| **Subject** | **Left CA** | **LCCA** | **LICA** | **LECA** | **Right CA** | **RCCA** | **RICA** | **RECA** | **Sum Score*** | **Rank** |
| --- | --- | --- | --- | --- | --- | --- | --- | --- | --- | --- |
| **1** | 0.89 | 0.87 | 0.74 | 0.56 | 0.77 | 0.94 | 0.74 | 0.87 | 4.72 | 19 |
| **2** | 0.91 | 0.80 | 0.88 | 0.41 | 0.80 | 0.92 | 0.72 | 0.75 | 4.49 | 21 |
| **3** | 0.94 | 0.91 | 0.89 | 0.92 | 0.93 | 0.89 | 0.94 | 0.91 | 5.46 | 4 |
| **4** | 0.94 | 0.88 | 0.87 | 0.74 | 0.94 | 0.92 | 0.91 | 0.84 | 5.15 | 9 |
| **5** | 0.95 | 0.94 | 0.92 | 0.92 | 0.96 | 0.93 | 0.94 | 0.93 | 5.58 | 2 |
| **6** | 0.57 | 0.58 | 0.12 | 0.62 | 0.61 | 0.77 | 0.32 | 0.00 | 2.40 | 23 |
| **7** | 0.88 | 0.89 | 0.84 | 0.84 | 0.90 | 0.83 | 0.84 | 0.82 | 5.07 | 12 |
| **8** | 0.92 | 0.88 | 0.86 | 0.87 | 0.90 | 0.88 | 0.89 | 0.86 | 5.24 | 8 |
| **9** | 0.73 | 0.92 | 0.93 | 0.89 | 0.95 | 0.70 | 0.86 | 0.51 | 4.82 | 17 |
| **10** | 0.83 | 0.71 | 0.69 | 0.77 | 0.76 | 0.82 | 0.79 | 0.79 | 4.59 | 20 |
| **11** | 0.92 | 0.92 | 0.82 | 0.70 | 0.94 | 0.89 | 0.81 | 0.84 | 4.99 | 14 |
| **12** | 0.92 | 0.62 | 0.57 | 0.90 | 0.75 | 0.90 | 0.85 | 0.90 | 4.73 | 18 |
| **13** | 0.93 | 0.94 | 0.86 | 0.79 | 0.91 | 0.86 | 0.85 | 0.79 | 5.08 | 11 |
| **14** | 0.95 | 0.93 | 0.96 | 0.93 | 0.97 | 0.93 | 0.95 | 0.91 | 5.61 | 1 |
| **15** | 0.93 | 0.95 | 0.94 | 0.94 | 0.97 | 0.84 | 0.89 | 0.88 | 5.44 | 6 |
| **16** | 0.91 | 0.80 | 0.71 | 0.75 | 0.82 | 0.84 | 0.90 | 0.83 | 4.84 | 16 |
| **17** | 0.78 | 0.82 | 0.38 | 0.72 | 0.76 | 0.87 | 0.29 | 0.78 | 3.85 | 22 |
| **18** | 0.86 | 0.91 | 0.81 | 0.89 | 0.90 | 0.84 | 0.66 | 0.88 | 4.99 | 15 |
| **19** | 0.96 | 0.76 | 0.81 | 0.66 | 0.80 | 0.91 | 0.97 | 0.94 | 5.05 | 13 |
| **20** | 0.94 | 0.90 | 0.89 | 0.92 | 0.93 | 0.92 | 0.92 | 0.89 | 5.45 | 5 |
| **21** | 0.95 | 0.93 | 0.92 | 0.91 | 0.94 | 0.92 | 0.95 | 0.88 | 5.51 | 3 |
| **22** | 0.88 | 0.89 | 0.83 | 0.86 | 0.90 | 0.84 | 0.87 | 0.82 | 5.10 | 10 |
| **23** | 0.88 | 0.90 | 0.86 | 0.90 | 0.91 | 0.84 | 0.89 | 0.86 | 5.24 | 7 |
| **Mean** | 0.89 | 0.85 | 0.79 | 0.80 | 0.87 | 0.87 | 0.82 | 0.80 | 4.93 |  |
| **sd** | 0.09 | 0.10 | 0.19 | 0.14 | 0.09 | 0.06 | 0.18 | 0.19 | 0.67 |  |

## **Additional file 1: Table S5. F_0.5**

**F-Beta, Beta = 0.5**

*** -** Sum Score of Left and Right CCA, ICA, and ECA. A score of 6 represents a perfect score.

| **Subject** | **Left CA** | **LCCA** | **LICA** | **LECA** | **Right CA** | **RCCA** | **RICA** | **RECA** | **Sum Score*** | **Rank** |
| --- | --- | --- | --- | --- | --- | --- | --- | --- | --- | --- |
| **1** | 0.90 | 0.89 | 0.91 | 0.62 | 0.85 | 0.87 | 0.89 | 0.82 | 4.99 | 9 |
| **2** | 0.92 | 0.83 | 0.83 | 0.52 | 0.84 | 0.75 | 0.91 | 0.80 | 4.64 | 14 |
| **3** | 0.86 | 0.88 | 0.80 | 0.83 | 0.86 | 0.83 | 0.87 | 0.78 | 4.99 | 10 |
| **4** | 0.82 | 0.64 | 0.76 | 0.75 | 0.79 | 0.76 | 0.80 | 0.77 | 4.49 | 16 |
| **5** | 0.93 | 0.91 | 0.91 | 0.87 | 0.92 | 0.92 | 0.90 | 0.90 | 5.40 | 1 |
| **6** | 0.71 | 0.52 | 0.20 | 0.73 | 0.67 | 0.74 | 0.44 | 0.00 | 2.63 | 23 |
| **7** | 0.79 | 0.84 | 0.80 | 0.73 | 0.83 | 0.77 | 0.73 | 0.72 | 4.59 | 15 |
| **8** | 0.76 | 0.67 | 0.71 | 0.68 | 0.71 | 0.70 | 0.77 | 0.69 | 4.24 | 18 |
| **9** | 0.82 | 0.88 | 0.80 | 0.89 | 0.88 | 0.89 | 0.83 | 0.61 | 4.89 | 11 |
| **10** | 0.94 | 0.86 | 0.85 | 0.85 | 0.91 | 0.93 | 0.91 | 0.86 | 5.27 | 5 |
| **11** | 0.77 | 0.80 | 0.85 | 0.77 | 0.88 | 0.81 | 0.64 | 0.58 | 4.45 | 17 |
| **12** | 0.81 | 0.69 | 0.79 | 0.88 | 0.85 | 0.79 | 0.83 | 0.72 | 4.70 | 13 |
| **13** | 0.90 | 0.95 | 0.94 | 0.87 | 0.96 | 0.84 | 0.83 | 0.70 | 5.14 | 8 |
| **14** | 0.95 | 0.89 | 0.87 | 0.83 | 0.89 | 0.95 | 0.94 | 0.90 | 5.36 | 3 |
| **15** | 0.92 | 0.90 | 0.89 | 0.89 | 0.92 | 0.83 | 0.90 | 0.86 | 5.27 | 4 |
| **16** | 0.81 | 0.93 | 0.84 | 0.82 | 0.94 | 0.81 | 0.78 | 0.64 | 4.82 | 12 |
| **17** | 0.77 | 0.63 | 0.66 | 0.63 | 0.74 | 0.70 | 0.58 | 0.82 | 4.02 | 22 |
| **18** | 0.78 | 0.78 | 0.60 | 0.67 | 0.72 | 0.72 | 0.69 | 0.76 | 4.23 | 19 |
| **19** | 0.92 | 0.78 | 0.90 | 0.78 | 0.87 | 0.89 | 0.93 | 0.88 | 5.16 | 7 |
| **20** | 0.90 | 0.84 | 0.90 | 0.86 | 0.89 | 0.89 | 0.89 | 0.83 | 5.21 | 6 |
| **21** | 0.92 | 0.96 | 0.91 | 0.84 | 0.93 | 0.87 | 0.90 | 0.91 | 5.39 | 2 |
| **22** | 0.72 | 0.66 | 0.61 | 0.72 | 0.69 | 0.61 | 0.72 | 0.81 | 4.13 | 21 |
| **23** | 0.72 | 0.72 | 0.66 | 0.72 | 0.72 | 0.73 | 0.71 | 0.64 | 4.19 | 20 |
| **Mean** | 0.84 | 0.80 | 0.78 | 0.77 | 0.84 | 0.81 | 0.80 | 0.74 | 4.70 |  |
| **sd** | 0.08 | 0.12 | 0.16 | 0.10 | 0.09 | 0.08 | 0.12 | 0.18 | 0.62 |  |

## **Additional file 1: Table S6. TPR**

**True Positive Ratio (TPR)**

*** -** Sum Score of Left and Right CCA, ICA, and ECA. A score of 6 represents a perfect score.

| **Subject** | **Left CA** | **LCCA** | **LICA** | **LECA** | **Right CA** | **RCCA** | **RICA** | **RECA** | **Sum Score*** | **Rank** |
| --- | --- | --- | --- | --- | --- | --- | --- | --- | --- | --- |
| **1** | 0.88 | 0.87 | 0.70 | 0.54 | 0.75 | 0.96 | 0.70 | 0.89 | 4.66 | 19 |
| **2** | 0.91 | 0.80 | 0.90 | 0.38 | 0.79 | 1.00 | 0.68 | 0.74 | 4.49 | 20 |
| **3** | 0.98 | 0.92 | 0.92 | 0.96 | 0.96 | 0.91 | 0.97 | 0.96 | 5.64 | 5 |
| **4** | 0.99 | 1.00 | 0.92 | 0.74 | 1.00 | 0.98 | 0.95 | 0.87 | 5.45 | 10 |
| **5** | 0.96 | 0.95 | 0.93 | 0.94 | 0.97 | 0.94 | 0.95 | 0.94 | 5.64 | 4 |
| **6** | 0.54 | 0.60 | 0.11 | 0.59 | 0.59 | 0.77 | 0.29 | 0.00 | 2.35 | 23 |
| **7** | 0.91 | 0.91 | 0.85 | 0.89 | 0.92 | 0.85 | 0.89 | 0.86 | 5.25 | 13 |
| **8** | 0.99 | 0.97 | 0.93 | 0.97 | 0.99 | 0.96 | 0.93 | 0.93 | 5.70 | 3 |
| **9** | 0.71 | 0.93 | 0.98 | 0.89 | 0.98 | 0.66 | 0.86 | 0.49 | 4.82 | 17 |
| **10** | 0.80 | 0.67 | 0.65 | 0.75 | 0.72 | 0.79 | 0.76 | 0.77 | 4.40 | 21 |
| **11** | 0.98 | 0.97 | 0.81 | 0.68 | 0.96 | 0.93 | 0.90 | 0.99 | 5.27 | 12 |
| **12** | 0.96 | 0.60 | 0.52 | 0.90 | 0.72 | 0.95 | 0.86 | 0.98 | 4.80 | 18 |
| **13** | 0.94 | 0.93 | 0.83 | 0.76 | 0.89 | 0.87 | 0.85 | 0.83 | 5.07 | 14 |
| **14** | 0.95 | 0.95 | 0.99 | 0.97 | 1.00 | 0.92 | 0.96 | 0.91 | 5.71 | 2 |
| **15** | 0.93 | 0.96 | 0.96 | 0.96 | 0.99 | 0.84 | 0.89 | 0.89 | 5.51 | 9 |
| **16** | 0.95 | 0.76 | 0.68 | 0.73 | 0.79 | 0.86 | 0.95 | 0.92 | 4.90 | 16 |
| **17** | 0.78 | 0.91 | 0.34 | 0.75 | 0.76 | 0.94 | 0.25 | 0.76 | 3.95 | 22 |
| **18** | 0.90 | 0.96 | 0.92 | 1.00 | 0.98 | 0.89 | 0.65 | 0.93 | 5.34 | 11 |
| **19** | 0.97 | 0.76 | 0.79 | 0.63 | 0.78 | 0.91 | 0.98 | 0.96 | 5.03 | 15 |
| **20** | 0.95 | 0.92 | 0.89 | 0.94 | 0.95 | 0.93 | 0.93 | 0.91 | 5.53 | 8 |
| **21** | 0.96 | 0.92 | 0.93 | 0.94 | 0.95 | 0.93 | 0.97 | 0.87 | 5.56 | 7 |
| **22** | 0.95 | 1.00 | 0.94 | 0.91 | 1.00 | 0.96 | 0.94 | 0.83 | 5.57 | 6 |
| **23** | 0.95 | 0.98 | 0.95 | 0.98 | 1.00 | 0.89 | 0.96 | 0.97 | 5.73 | 1 |
| **Mean** | 0.91 | 0.88 | 0.80 | 0.82 | 0.89 | 0.90 | 0.83 | 0.83 | 5.06 |  |
| **sd** | 0.10 | 0.12 | 0.22 | 0.16 | 0.12 | 0.08 | 0.20 | 0.21 | 0.75 |  |

## **Additional file 1: Table S7. Summary of Statistical tests**

Results of two sampled t-tests between segmentation scores. * - p < 0.0025

|  | **DSC** | **MCC** | **F_2_** | **F_0.5_** | **TPR** |
| --- | --- | --- | --- | --- | --- |
| **Bifurcation v Branches** | 0.007 | 0.009 | 0.020 | 0.009 | 0.051 |
| **CCA v ICA** | 0.135 | 0.143 | 0.045 | 0.578 | 0.035 |
| **CCA v ECA** | 0.032 | 0.029 | 0.032 | 0.065 | 0.051 |
| **ICA v ECA** | 0.635 | 0.563 | 0.981 | 0.250 | 0.798 |

## **Additional file 1: Table S8. Comparison between original and proposed DeepMedic Configurations before and after post-processing.**

N=20 Bifurcations, all regions

| **Before Post-Processing** | |
| --- | --- |
| **Configuration** | **DSC** |
| Original | 0.685 |
| Modified | 0.811 |
| **After Post-Processing** | |
| **Configuration** | **DSC** |
| Original | 0.833 |
| Modified | 0.875 |

## **Additional file 1: Figure S1. Example Segmentations with poor performance**


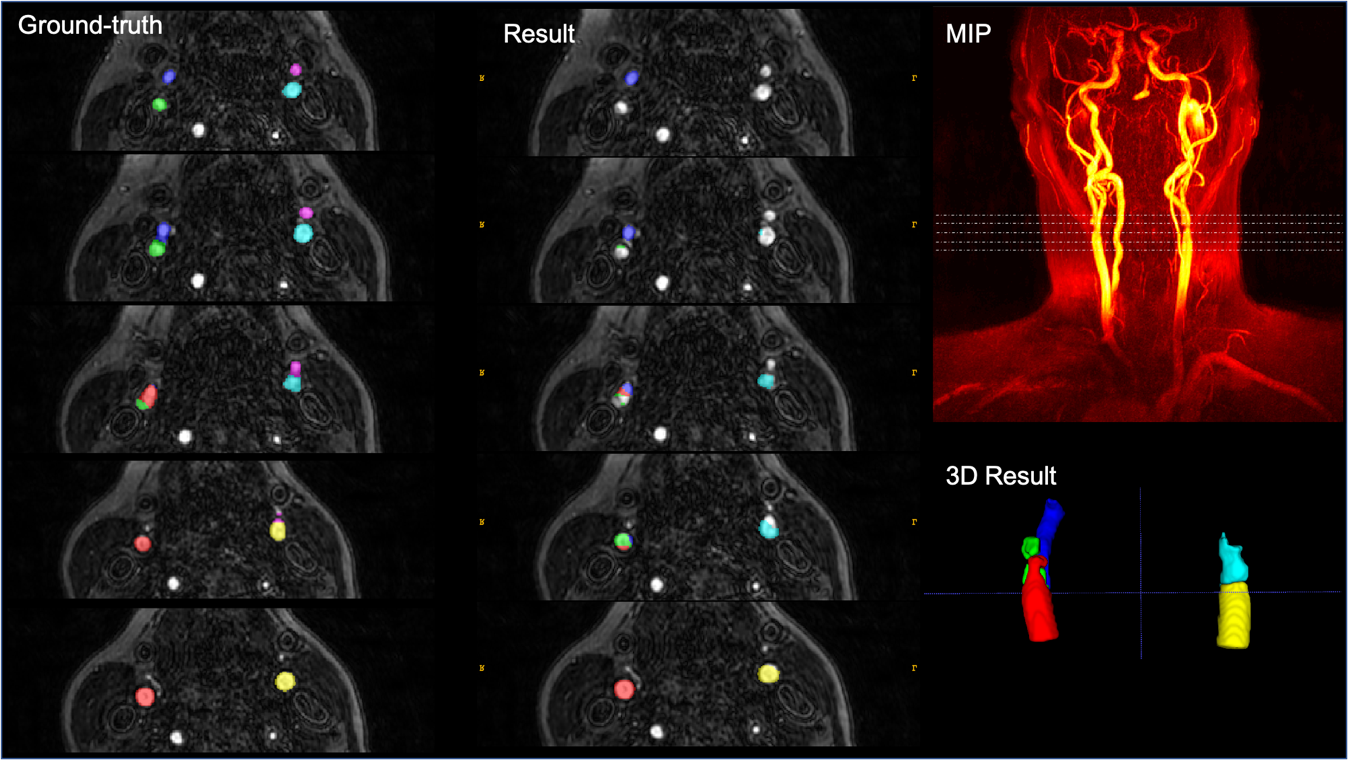


Example subject (#6) with unacceptably poor segmentation performance.

DSC scores for this subject were: Left 0.63, LCCA 0.55, LICA 0.15, LECA 0.67, Right 0.64, RCCA 0.75, RICA 0.37, RECA 0.
